# Introducing a triage and Nurse on Call model in primary health care – a focus group study of health care staff’s experiences

**DOI:** 10.1186/s12913-023-10300-5

**Published:** 2023-11-24

**Authors:** Maria Gelin, Berit Gesar, Ann-Sofie Källberg, Anna Ehrenberg, Catharina Gustavsson

**Affiliations:** 1 Center for Clinical Research Dalarna, Uppsala University, Nissers väg 3, SE-79182 Falun, Sweden; 2 School of Health and Welfare, Dalarna University, Falun, SE-79188 Sweden; 3 Department of Public Health and Caring Sciences, Uppsala University, Uppsala, Sweden

**Keywords:** Accessibility of health services, Focus groups, Nurse on Call, Primary health care, Qualitative content analysis, Triage

## Abstract

**Background:**

With the increased demand for health care services and with simultaneous staff shortages, new work models are needed in primary health care. In November 2015, a Swedish primary health care centre introduced a work model consisting of a structured patient sorting system with triage and Nurse on Call. The aim of this study was to describe the staff’s experiences of introducing the triage and Nurse on Call model at the primary health care centre.

**Methods:**

Five focus group discussions with staff (n = 39) were conducted 4 years after the introduction of the work model. Groups were divided by profession: medical secretaries, nursing assistants, physicians, primary health care nurses, and registered nurses. The transcribed text from the discussions was analysed using qualitative inductive content analysis.

**Results:**

The analysis generated one overarching theme: The introduction of triage and Nurse on Call addresses changed preconditions in primary health care, but the work culture, organization, and acquisition of new knowledge are lagging behind. The overarching theme had five categories: (1) Changed preconditions in primary health care motivate new work models; (2) The triage and Nurse on Call model improves teamwork and may increase the quality of care; (3) Unclear purpose and vague leadership make introducing the work model difficult; (4) Difficulties to adopt the work model as it challenges professional autonomy; and (5) The triage and Nurse on Call model requires more knowledge and competence from nurses in primary health care.

**Conclusions:**

This study contributes with knowledge about implications of a new work model in primary health care from the perspective of health care staff. The work model using triage and Nurse on Call in primary health care was perceived by participants to increase availability and optimize the use of resources. However, before introduction of new work models, it is important to identify barriers to and facilitators for successful improvements in the local health care context. Additional education for the health care staff is important if the transition is to be successful. Complementary skills and teamwork, supported by a facilitator seems important to ensure a well-prepared workforce.

**Supplementary Information:**

The online version contains supplementary material available at 10.1186/s12913-023-10300-5.

## Background

Health care staff are in short supply in Sweden and many other countries. Increased demand for health care and a shortage of staff means that new work models in primary health care (PHC) are needed to ensure patients’ access to safe, cost-effective and coordinated care [1–3]. Optimizing the use of health care staff’s competences in PHC can be achieved by referring patients to the health care professional who is best qualified for the work task [4, 5].

The aim of classifying patients, and services, according to triage [6, 7] and thus optimizing the use of the health care professionals’ competences [4, 5] is to increase the effective use of resources and the quality of health care. Triage is used in emergency care to assess the level of urgency and determine the order in which patients should be attended to [8, 9]. Nurses in telephone counselling apply triage based on computerized decision support tools [10–12].

In Swedish PHC, a physician is usually the first point of contact if the patient needs same-day medical attention. One Swedish PHC introduced a work model consisting of a structured patient-sorting system based on triage and work in teams of health care professionals. The main findings were an increase in access to PHC for the patients and a more efficient use of the staff’s competence [13]. There are studies of task shifting from physicians to nurses [14–16] and of nurses taking over work tasks from physicians in PHC [5]. Several studies have reported that physiotherapists are the preferred first point of contact for people seeking PHC for musculoskeletal complaints [17–19]. In some studies nurses have been the first point of contact in PHC walk-in centres [20] and after-hours PHC [21]. These studies have shown that registered nurses (RNs) can independently fill gaps and often replace physicians, but few studies have been made where the nurse on call is the preferred first point of contact for patients seeking PHC for acute complaints [22].

In summary, there are reasons to believe that triage and optimizing the use of the health care staff’s competences could increase the quality of health care and optimize the use of resources. In 2015, a Swedish PHC centre introduced a work model consisting of a structured patient sorting system using triage and Nurse on Call. The purpose was to increase accessibility and health care quality in PHC. Patients who needed same-day assessment at the PHC centre were allocated to a nurse on call rather than a physician on call. The aim of this study was to describe the staff’s experiences of introducing the triage and Nurse on Call model at the PHC centre.

## Methods

### Design

This study has a descriptive design using qualitative inductive content analysis [23] of focus group discussions [24] with PHC staff. The reporting follows the Standards for Reporting Qualitative Research (SRQR) [25].

### Setting and participants

This study was undertaken at a publicly owned PHC centre in Sweden. A large percentage of the patients in the catchment area (n = 14,200) had lower socioeconomic status and higher unemployment compared with the average residential area in Sweden, and represented diverse ethnic groups and religions. The PHC centre had about 70 employees. The management consisted of a head manager and three under-managers. The staff had different ethnic backgrounds and it was estimated that the employees together spoke about 25 languages. Several physicians were on short-term contract as there was a shortage of permanently employed physicians. By contrast, almost all of the primary health care nurses (PHCNs) had worked at the PHC centre for many years and had extensive professional experience as specialist nurses with PHC training [26]. During the summers, medical students, who still were undergoing training, worked at the PHC centre; in this article they are referred to as “physicians’ assistants”.

### Before introduction of the work model

Before the model was introduced, patients used to contact the PHC centre by telephone to get advice or book an appointment. The telephone counselling was handled by PHCNs and RNs, hereafter called ‘telephone nurses’. They did not use any standardized assessment methods in telephone counselling. Physicians provided the first point of contact for patients who needed same-day assessment. Patients who did not need same-day assessment care often ended up on a waiting list because there were not enough physician appointments available. The RNs assisted the physicians with patients who needed treatment on the same day. The PHCNs had a separate consultation area for planned patient visits for treating wounds, rashes and sore throats, inserting or replacing catheters, and administering injections.

### After introduction of the work model

The new work model for implementation of triage and Nurse on Call was a development project introduced at the PHC centre in November 2015. Prior to its introduction, a project description outlining the model’s purpose, goals and implementation plan was prepared. The model contained four major changes compared with the previous work model: (1) Telephone nurses were to use two decision support tools [10, 13] that provided guidance for sorting patients based on assessment of the degree of urgency and also based on which health care professional was needed. (2) Patients who contacted the PHC centre and were assessed as needing same-day assessment were allocated to a nurse on call as first point of contact, instead of a physician. (3) Vital signs (pulse, blood pressure, saturation, temperature and respiratory rate) in all patients who needed same-day assessment or immediate care were measured by nurse assistants [27]. (4) A physician was available for referrals from the nurse on call, if needed.

**Fig. 1:**
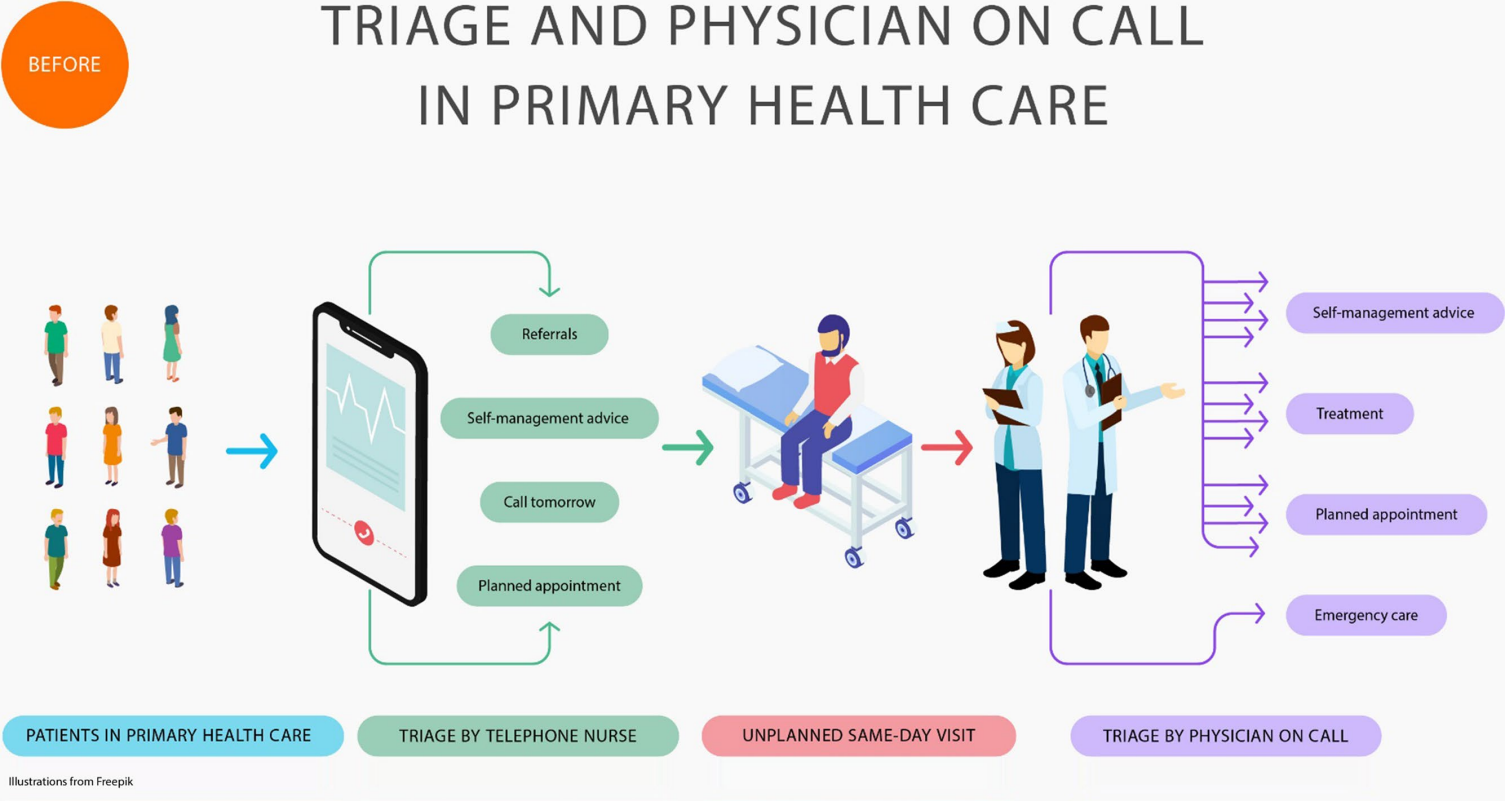
Description of the patient flow from telephone counselling to same-day assessment at the primary health care (PHC) centre before introduction of the work model based on triage and the Nurse on Call model

**Fig. 2:**
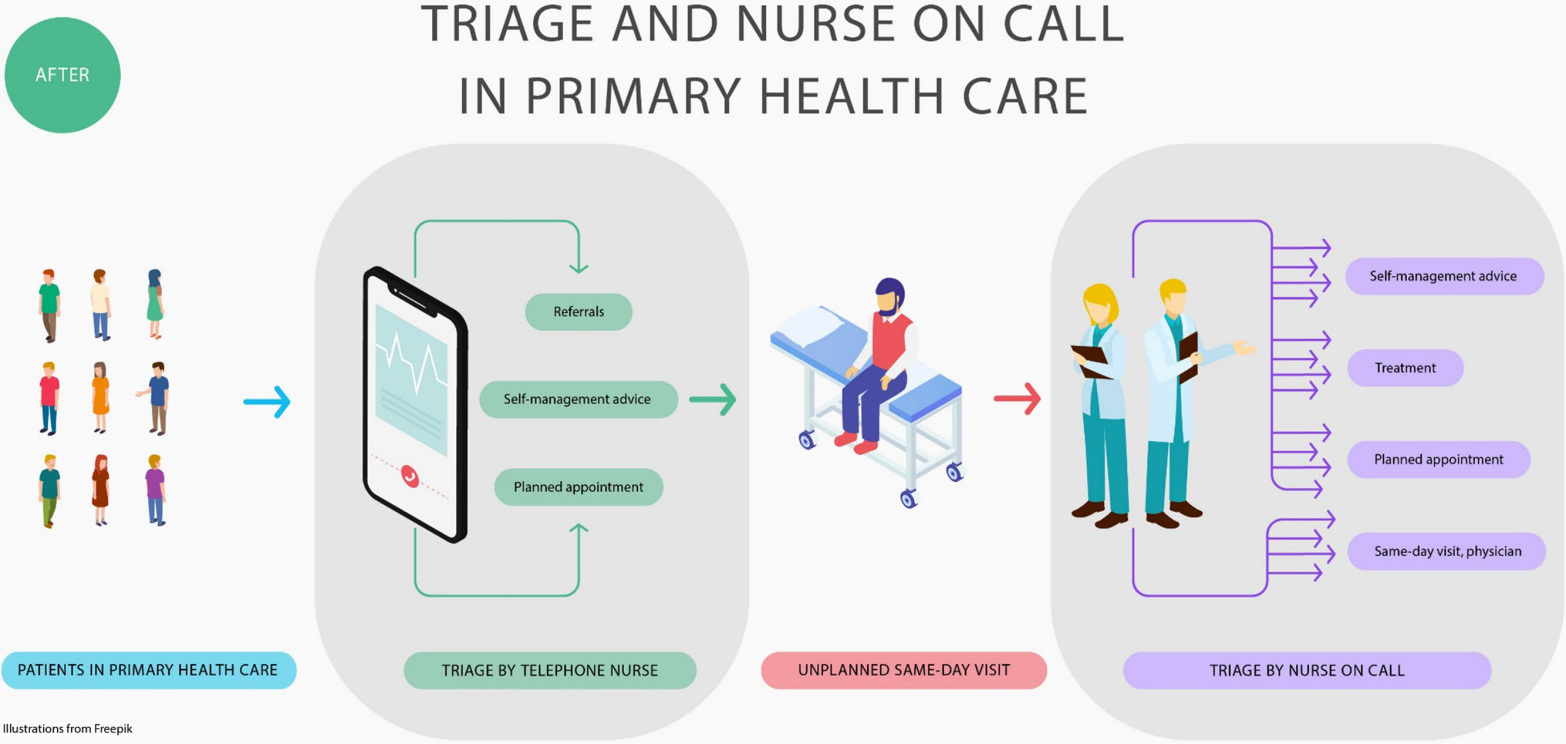
Description of the patient flow from telephone counselling to same-day assessment at the primary health care (PHC) centre after introduction of the work model based on triage and the Nurse on Call model

All staff received a short training on the new work model and the importance of taking vital signs. Telephone nurses received special training in using the two decision support tools [10, 13]. The RNs and some of the PHCNs were introduced to work as a nurse on call. One PHCN was given the role of facilitator to support the implementation process. Descriptions of the patient flow in the previous and the changed work model are presented in Figs. 1 and 2.

### Procedure for data collection

The manager of the PHC centre was contacted by the first author, and approved the study. All medical secretaries, nurse assistants, PHCNs, physicians and RNs had been introduced to, and had worked with, the work model consisting of triage and Nurse on Call and were therefore invited to participate in focus group discussions on this model. Table 1 presents the background characteristics of the participants. Physiotherapists and specialist nurses who worked with preventive health care for children were not affected by the work model and were therefore not included in the study.

**Table 1:** Characteristics of the participants (n = 39) in focus group discussions

	Focus group 1 PHCNs (n = 6)	Focus group 2 Medical secretaries (n = 9)	Focus group 3 Nurse assistants (n = 7)	Focus group 4 Physicians (n = 10)	Focus group 5 RNs (n = 7)
Women, n	6	9	7	4	7
Men, n	–	–	–	6	–
Age, yrs, mean (range)	58 (46–64)	45 (21–66)	46 (36–61)	46 (28–60)	44 (32–63)
Years of professional experience, mean (range)	28 (15–42)	16 (0.5–46)	17 (6–42)	15 (2–25)	16 (4–39)
Years of working at the PHC centre, mean (range)	12 (1–30)	12 (0.5–39)	7 (0.5–15)	5 (0.5–14)	2 (0.5–6)

PHC: Primary health care; PHCN: primary health care nurse; RN: registered nurse

Verbal and written information about the study was provided to the staff prior to the focus group discussions. Written informed consent was obtained from all participants. Information about the participants’ profession, gender and age, and number of years in the profession was noted. The focus group discussions [24] were conducted during working hours in September 2019 in a conference room at the PHC centre. The participants were allocated time in their work schedule for the focus group interview, and participation was voluntary. The participants were divided into five focus groups based on their profession. The focus groups were conducted by two of the researchers (BG and ASK). Both had previous experience in conducting semi-structured qualitative interviews and had no prior relationship with the participants. One researcher (BG) moderated the discussion and another author (ASK) assisted and took notes of any interactions and non-verbal communication in the group. A semi-structured interview guide was used which was developed specifically for the study (Supplementary file 1). It consisted of two main questions:

- Can you tell us about your experiences of work using the work model based on triage and Nurse on Call?
- What would need to be done differently if a similar project were to be initiated at another PHC centre?

The moderator added probing questions and summarized the discussions to check that they had been interpreted correctly. Discussions ranged from 42 to 60 min. Focus group discussions were audio-recorded and transcribed verbatim.

### Data analysis

Qualitative content analysis with an inductive approach inspired by Elo & Kyngas was used [23]. All authors read the transcribed focus group discussions and participated in the analytical coding process. The authors are licensed health care professionals, two with previous experience of working in PHC and three with experience of triage in emergency care. To make sense of data the first author (MG) listened to the audio-recordings and read the transcripts of the focus group discussions several times. The first and last author (MG and CG) performed the preliminary data extraction. Meaning units in the text relating to the research question were identified, condensed and coded. The codes were grouped into subcategories and were further aggregated to broader categories. Thereafter, all authors participated in elaborating the preliminary analysis and discussed the emerging result. The authors moved back and forth between meaning units, codes, subcategories and categories until there was consensus in the research group.

## Results

The analysis of the focus group discussions generated one overarching theme : The introduction of triage and Nurse on Call addresses changed preconditions in PHC, but the work culture, organization and acquisition of new knowledge are lagging behind. The overarching theme comprised five categories as displayed in Table 2. The content of the categories is outlined below, with quotes in italics.

**Table 2:** Overview of the results of the content analysis of the focus group discussions

Categories	Subcategories	Examples of meaning units/Quote
Changed preconditions in PHC motivate new work models	Patients’ expectations of health care have changed The resources in PHC, i.e. staff and premises, are not adapted to new conditions	One problem is that our patients do not know their diseases and symptoms, instead they seek emergency care at the PHC centre. There are so many changes that just arise at this PHC centre. For us PHCNs, it feels like we remain behind on the platform when the train passes by.
The triage and Nurse on Call model improves teamwork and may increase the quality of care	Triage and Nurse on Call increase patient safety Triage and Nurse on Call increase patients’ accessibility to health care	When you follow a decision support tool it feels like increasing patient safety. We work closer together and across boundaries, especially between physicians and RNs.
Unclear purpose and vague leadership make introducing the work model difficult	Unclear purpose at introduction Vague leadership and insufficient organizational support	Everything was fine until things [the work model] were tightened up one year ago. Since then we PHCN no longer can book appointments to physician on call although we want to. When we try to say what we think about the work model with triage and Nurse on Call the manager literally says, “We will not turn back. Before, when we had no real rules, you did what you thought was right.
Difficulties to adopt the work model as it challenges professional autonomy	The approach of optimizing the use of the health care professionals’ competences is questioned because of the strong tradition of work in PHC being physician-centred The changed working method questions how nurses have traditionally worked in PHC	Sometimes you set up a direct consultation with the physician on call … to spare the patient the nurse on call consultation, which doesn’t lead to anything anyway. Problems arise when some of the telephone nurses book as they wish instead of following the decision support tools.
The triage and Nurse on Call model requires more knowledge and competence from nurses in PHC	Lack of correspondence with nurses’ experience, knowledge and competence Expanded responsibility and more advanced tasks for nurses	When you advise patients over the phone, you learn to listen between the lines and to listen to your own gut feeling. When we introduced the work model, it was important to think in a different way. So we had a facilitator at the PHC centre available every day. She was experienced in working as a nurse on call. Today, that kind of support is no longer available. Registered nurses and PHCNs need to get more education before starting with the new work model where they’re expected to take more responsibility.

PHC: primary health care; PHCN: primary health care nurse; RN: registered nurse

### Changed preconditions in PHC motivate new work models

Participants in all focus group discussions pointed out that patients’ expectations and reasons for seeking PHC had changed during recent years. This meant that today they seek same-day care for both emergency and minor complaints with mild symptoms that should be cured with self-care. Especially the physicians and RNs highlighted that the work model is suitable when patients contact the PHC centre with minor complaints, expecting same-day attention.

One problem is that our patients have low knowledge of diseases and symptoms. Instead they seek emergency care at the PHC centre. (physician)

Physicians, PHCNs and RNs expressed that an important benefit of the work model was that the telephone nurse could refer patients to the most suitable health care professional. They perceived that the nurse on call, instead of physicians, should assess and treat patients with less complex conditions.

Overall, the participants expressed that the structured sorting of patients according to the triage and Nurse on Call model was a good solution to meet patients’ needs and to make priority for patients with the greatest needs for care. They suggested that it might be possible to develop this further, for example by increasing teamwork, involving more health care professions, getting more knowledge about emergency care, and strengthening nurse assistants in their role. However, the work model was perceived as troublesome by some of the PHCNs. They described that the PHC centre had changed into a ‘light emergency ward’.

There are so many changes that just arise at this PHC centre. For us PHCNs, it feels like we remain behind on the platform when the train passes by. (PHCN)

The physicians expressed that the work model was sometimes undeservedly criticized by other health care staff at the PHC centre. This was because other staff did not understand that the real problem was shortage of staff and not the work model.

### The triage and Nurse on Call model improves teamwork and may increase the quality of care

The participants perceived that the work model increased the quality of health care. Physicians and RNs pointed out that the work model entailed higher accessibility and patient safety compared with the previous way of working.

When you follow a decision support tool it feels like increasing patient safety. (RN)

Simultaneously the physicians on call perceived that their workload had decreased since the new model of triage and Nurse on Call had been introduced. The work model stipulated that the patient should receive an initial assessment by the RN before seeing the physician. This assured that only patients who needed medical care from a physician on call would be referred. However, it also became obvious that telephone nurses’ lack of compliance with the work model increased the workload for the other staff. This was particularly evident for physicians who were available for referrals.

Nurse assistants, RNs and medical secretaries emphasized that the work model had contributed to more teamwork and better collaboration between the professions. The nursing assistants also perceived that their new role in the team had increased their status. Both nursing assistants and RNs expressed that it had broadened and developed their work and made it more enjoyable since they could make use of their skills and work in a team.

We work closer together and across boundaries, especially between physicians and RNs. (medical secretary)

Another benefit of the work model was that it entailed optimizing the use of the health care professionals’ competences. The physicians perceived that it increased access to care for more patient groups, such as people with chronic diseases and multimorbidity.

### Unclear purpose and vague leadership make introducing the work model difficult

The telephone nurses described that the decision support tools worked well in the beginning. This was because they (i.e. the telephone nurses) still felt free to deviate from the triage and Nurse on Call path. The RNs appreciated that they were allowed to take more responsibility. However, the RNs and nurse assistants perceived that they had to adhere strictly to the work tasks outlined for them by the work model.

Before, when we had no real rules, you did what you thought was right. (PHCN)

The focus group discussions revealed that PHCNs had been allowed by the manager to opt out of the work task to be nurse on call, but the RNs and nursing assistants were not allowed to opt out of any work tasks. The PHCNs expressed that the high workload for nurse on call required stricter adherence to the decision support tools from the telephone nurses. At the same time, PHCNs disliked being forced to adhere to the decision support tools when they themselves worked as telephone nurses. The PHCNs and RNs disapproved of the new work model because they perceived that there were constant changes over which they had no control. They perceived that the work model had been introduced without discussion or consultation and consequently there was no mutual understanding among members of staff.

Everything was fine until things [the work model] were tightened up one year ago. Since then we PHCN no longer can book appointments to physician on call although we want to. (PHCN)

The PHCNs expressed frustration because the manager did not pay attention to their hesitation or consult them for suggestions based on their solid experience. They generally also expressed being overwhelmed by written information.

When we try to say what we think about the work model with triage and Nurse on Call the manager literally says, “We will not turn back. (PHCN)

The triage and Nurse on Call model had given rise to conflict at the PHC centre. The PHCNs perceived that the head manager was solely responsible for introducing the work model and meant that no other profession had been involved in the decision or process. The physicians perceived that there was a conflict between the PHCNs and the RNs, and that the sometimes poor adherence to the work model negatively affected their workload and contributed to an ineffective work situation.

### Difficulties to adopt the work model as it challenges professional autonomy

Although the staff expressed many positive experiences, there were also expressions of lacking acceptance and adherence to the work model. Always using the decision support tool and booking appointments with the nurse on call for patients in need of same-day attention was considered unnecessary by some of the PHCNs and RNs. They perceived that the nurse on call was redundant, because often this nurse was unable to perform the initial assessment. Therefore, telephone nurses booked some patients to be directly seen by a physician. They expressed that, based on their professional experience, they knew when a patient needed a physician´s consultation.

Sometimes you set up a direct consultation with the physician on call … to spare the patient the nurse on call consultation, which doesn’t lead to anything anyway. (RN)

Telephone nurses expressed that the lack of available physician’s appointments was one reason for not adhering to the decision support tools. Instead, they set up direct appointments with the physician on call who was overbooked. They based their decision that a patient needed to see a physician on their own experience, and thereby deviated from the new work model. As a result, same-day physician’s appointments were rescheduled to planned appointments.

Problems arise when some of the telephone nurses book as they wish instead of following the decision support tools. (physician)

It was a common view among the nurse assistants, PHCNs and RNs that having patients meet several health care professionals during the course of one appointment was a waste of resources. This entailed that the patient had to repeat their symptoms to several medical personnel. The RNs and medical secretaries also perceived that some patients were disappointed when they understood that they were to meet a nurse on call instead of a physician. They were frustrated when they had to spend time explaining to patients why they were being seen by a nurse rather than a physician. According to the RNs and medical secretaries, some patients and even colleagues who had immigrated from other countries were not aware of the competence of Swedish nurses. Especially the medical secretaries pointed out that telephone nurses should explain the work model to patients.

Another comment suggesting difficulties to adopt the work model was made by PHCNs, who described that using the model meant being underutilized as they did not utilize their professional experience-based knowledge when obliged to follow the decision support tools. In their opinion, the decision support tools should be revised and their capability to determine what appointment type was needed should be respected.

### The triage and Nurse on Call model requires more knowledge and competence from nurses in PHC

The RNs and PHCNs expressed that the PHC centre’s most difficult work task is the telephone nurse’s task and telephone counselling. Their opinion was that the triage and Nurse on Call model using the decision support tools made the telephone nurse’s work more straightforward while at the same time making it more complex. They said that they could not fully rely on the decision support tools during telephone consultations, for example when they suspected that a patient was exaggerating their symptoms.

When you advise patients over the phone, you learn to listen between the lines and to listen to your own gut feeling. (RN)

However, according to the PHCNs, the new duties of the nurse on call were acute and emergency care, which they were not qualified for. They regarded themselves as a forgotten professional group with competence that was no longer needed.

The physicians and RNs said that experienced staff are vital for good collaboration and for keeping the team’s workload reasonable. The nurses on call described feeling unsure when no physicians were available to see patients with acute conditions that RNs are not qualified to handle. By contrast, the physicians expressed that the nurses on call needed to attain the necessary skills and experience to make the model work.

Registered nurses and PHCNs need to get more education before starting with the new work model where they’re expected to take more responsibility. (physician)

The support from the facilitator was considered crucial for successful implementation. According to the RNs, such support, but also training and continuing education, is important for increasing RNs’ and PHCNs’ competence and making them confident to handle more of the less complicated conditions in PHC.

When we introduced the work model, it was important to think in a different way. So we had a facilitator at the PHC centre available every day. She was experienced in working as a nurse on call. Today, that kind of support is no longer available. (RN)

## Discussion

According to our results the new work model was experienced by staff as both positive and negative. The health care staff perceived that the work model largely met the objectives to increase availability and optimize the use of resources. The work model was also perceived to contribute to more teamwork and better collaboration between the professions. However, the participants identified several obstacles to successful implementation. Major obstacles included limited understanding of the purpose, difficulties in phasing out the previous work model, and lacking knowledge to manage new work tasks. All focus groups, especially the physicians and medical secretaries, perceived that telephone nurses’ compliance with the decision support tools was essential for this model to work.

The findings of this study indicate that there is a need for staff to understand the purpose of change before such change is introduced, and that implementation strategies were perceived as deficient. Before a work model is implemented, both management and staff need to agree on why the organization needs to change and what benefits such change might bring to the patients [28]. Greenhalgh et al. [29] and McMullen et al. [30] highlight that clear leadership and a clear purpose are cornerstones in adapting to changes in an organization. Participation, information and knowledge of changed methods increase the conditions for staff adaptation [29, 30]. Unclear purpose and leadership was mentioned as one reason why the current work model based on the triage and Nurse on Call model was perceived as not having reached its full potential four years later.

This study reveals that the RNs and PHCNs had a key role when implementing this new work model. Their work task as telephone nurses and nurses on call implied that they were the most appropriately qualified to perform this task. Their expected education could correspond to a nurse practitioner [31] who independently assesses and treats patients in PHC [32, 33]. In the present case, more education and staff participation in the implementation process would ensure a well-prepared nursing workforce [20, 31]. A framework and national regulations have also been shown to be important before introducing a new role as advanced practice nurse in PHC [34].

Fear of not having sufficient knowledge is likely one reason why PHCNs felt resistance to change in their professional roles. Other reasons may include fear of loss of professional identity. Health care professionals need to adapt and change behaviours to meet the needs of future health care [3].

Previous studies have shown that assessment of the proper level of care and self-management advice for patients are areas that need to be strengthened. Further education for telephone nurses can therefore lead to a decrease in the number of inappropriate health care visits and also of dissatisfied patients [35, 36].

Most of the health care staff said that the work model promoted teamwork and made work more pleasant. RNs, nurse assistants and medical secretaries experienced that their work had become more enjoyable, since they could make better use of their skills. Also, the physicians appreciated working in a team, as their workload was reduced, and they had more time for older patients and patients with complex needs. At the same time, physicians perceived that their workload was dependent on the skills of the nurse on call.

A Cochrane review has identified issues that influence PHC to have nurses conduct work tasks typically handled by physicians. The review concludes that both collaboration and work shifting between physicians and nurses increase the quality of health care as well as access to care [37]. Another study reported that lack of collaboration sometimes hindered health care staff from doing their best for the patient [38].

Most of the focus groups in this study considered the new work model to have benefits and to contribute to enhanced collaboration between health care professions. However, the PHCNs did not share this view. For them, the new work model implied that their skills were no longer acknowledged in the PHC centre. Insecurity in managing specific skills might reflect a fear of compromising professional identity. This could be a reason why PHCNs in the present study expressed resistance to change in their professional roles. Participants also expressed that patients often expect to see a physician when they contact the PHC. The phenomenon has been confirmed in other studies [37]. However, some studies have shown that patients are satisfied with being assessed and treated by a physiotherapist [39] or by an advanced practice nurse [40] in PHC.

The introduction of the work model was not a research intervention but an initiative from the manager at the PHC centre. The lack of implementation strategies proved to be a barriers expressed by the participants. There are plenty of challenges in developing work models to increase accessibility and offer individuals health care on equal terms [2]. The findings of this study suggest that work models involving triage and nurses on call may contribute to increase health care quality and optimize the use of resources. However, before implementation, it is important to identify barriers to and facilitators for successful improvements in the local health care context. Contextual determinants such as mutual agreement about the purpose, as well as the management and staff’s skills are important intervention components, which confirms the findings of our study [41, 42].

There are several strengths and limitations of this study. The decision to perform the focus group discussions based on professions instead of having mixed-staff groups may have influenced the results. Focus group discussions were chosen in order to generate insights into group dynamics and achieve maximum interaction between participants. This choice was considered to create a less censored discussion and provide deeper understanding [24]. Focus group discussions in professional groups have been stated to promote interaction and information exchange among the participants and allow them to produce rich data [23]. To ensure credibility two researchers who were not known to the PHC staff performed the focus group interviews (BG, ASK).

This study is retrospective, which may have affected the credibility and made the results uncertain, as the focus group discussions took place four years after introduction of the work model [43]. On the other hand, it can take several years before a new way of working is fully integrated. This is because introducing new work models requires many years before they are fully implemented in health care. There is also a risk that dominant participants may hinder other participants from expressing their views [24].

To ensure confirmability, a semi-structured interview guide was used in order that all aspects of the research question would be covered. This study was conducted at a single PHC centre in Sweden, which limits the transferability of our findings. Transferability is also limited as the organization of health care in the present health care region may differ from that in other health care regions. The credibility was enhanced in the data analysis; meaning units were identified and coding was done by the first author, followed by several discussions among all of the researchers, where subcategories, categories and finally one overaching theme emerged [23].

## Conclusion

This study contributes with knowledge about implications of a new work model in primary health care from the perspective of health care staff. The work model using triage and Nurse on Call in primary health care was perceived by participants to increase availability and optimize the use of resources. However, before introduction of new work models, it is important to identify barriers to and facilitators for successful improvements in the local health care context. Additional education for the health care staff is important if the transition is to be successful. Complementary skills and teamwork, supported by a facilitator seems important to ensure a well-prepared workforce.

### Electronic supplementary material

Below is the link to the electronic supplementary material.

Supplementary Material 1: Interview guide for the focus groups.

## Data Availability

The datasets are not publicly available. All analysed interview data are available from the corresponding author on reasonable request.

## List of abbreviations

PHC: primary health care
PHCN: primary health care nurse
RN: registered nurse
