# Supplementary material for: Introducing a triage and Nurse on Call model in primary health care – a focus group study of health care staff’s experiences
Source: BMC Health Serv Res. 2023 Nov 24;23:1299. doi: 10.1186/s12913-023-10300-5 (PMC10675943; doi:10.1186/s12913-023-10300-5)
Supplement: Supplementary file 1 — Supplementary Material 1: Interview guide for the focus groups. [file 12913_2023_10300_MOESM1_ESM.docx]

Supplementary file 1. The interview guide

| The interview guide used for the focus group discussions | |
| --- | --- |
| 1. | Can you tell us about your experiences of work using the work model based on triage and Nurse on Call? |
| 2. | What would need to be done differently if a similar project were to be initiated at another PHC centre |
